# Patients’ Satisfaction Regarding Oral Healthcare Services in the North-East Region of Romania: A Preliminary Questionnaire Survey

**DOI:** 10.3390/healthcare12121195

**Published:** 2024-06-13

**Authors:** Silviu Catalin Tibeica, Dragoș Ioan Virvescu, Iulian Costin Lupu, Dana Gabriela Budala, Ionut Luchian, Andreea Tibeica, Zinovia Surlari, Elena Mihaela Carausu

**Affiliations:** 1 Department of Health Management, Faculty of Dental Medicine, “Grigore T. Popa” University of Medicine and Pharmacy, 700115 Iasi, Romania; 2 Department of Fixed Prosthodontics, Faculty of Dental Medicine, “Grigore T. Popa” University of Medicine and Pharmacy, 700115 Iaşi, Romania; 3 Department of Dentures, Faculty of Dental Medicine, “Grigore T. Popa” University of Medicine and Pharmacy, 700115 Iaşi, Romania; 4 Department of Periodontology, Faculty of Dental Medicine, “Grigore T. Popa” University of Medicine and Pharmacy, 700115 Iasi, Romania; 5 Department of Implantology, Faculty of Dental Medicine, “Grigore T. Popa” University of Medicine and Pharmacy, 700115 Iași, Romania

**Keywords:** oral health, dental management, quality of life, patient satisfaction, quality management, dental services

## Abstract

This research addresses a gap in the literature by conducting a comprehensive analysis of patients’ level of satisfaction with dental care. Methods: By combining quantitative and qualitative survey methods with a PSQ, this study aims to augment ongoing initiatives to enhance dental patients’ experiences by painting a more comprehensive depiction of patients’ level of satisfaction. Results: When asked about their overall level of satisfaction 77.1% of the patients said that they received excellent services from office personnel and 72.2% said they trust their doctors. Conclusions: Assessing patient satisfaction in the realm of dental service quality is crucial for enhancing service quality and accuracy, which would benefit both patients and dentists and, ultimately, improve public health.

## 1. Introduction

Defining and measuring satisfaction is a challenging task due to its complexity. It is a psychological phrase that can be assessed via time and personal experiences [1]. It indicates the extent to which expected objectives have been achieved. Satisfaction includes cognitive and emotional aspects and is influenced by past experiences, expectations, and social connections [2,3]. Recently, there has been a growing emphasis on patient-centered care in the healthcare industry, emphasizing the significance of comprehending and improving patient happiness. Dental treatments, being a crucial part of healthcare, must meet these expectations; thus, high-quality dental services are essential for maintaining patient health, satisfaction, and general well-being [4,5].

The quality of dental care has a direct impact on patients’ oral health results and also plays a significant role in shaping their perception of the service provided, which, in turn, affects their loyalty and the probability of referring the service to others [6].

Although there is agreement on the significance of patient satisfaction in dental care, the research indicates a lack of thorough comprehension regarding the aspects that influence it [7]. Prior research has mostly concentrated on clinical results and the technical proficiency of dental practitioners, frequently neglecting aspects like interpersonal communication, service availability, and the dental practice environment [8,9].

Patient satisfaction is a critical indicator of healthcare quality and plays a vital role in the evaluation of medical services. It reflects patients’ perceptions of their healthcare experiences and is influenced by various factors, including the quality of care provided, the effectiveness of communication, and the interpersonal skills of healthcare providers [10].

High levels of patient satisfaction are associated with better adherence to treatment plans, improved clinical outcomes, and increased patient loyalty to healthcare providers and institutions [11]. Conversely, low levels of satisfaction can lead to poor health outcomes, reduced compliance with medical advice, and decreased utilization of healthcare services [12].

The relationship between patients and their primary care physicians is particularly important in determining patient satisfaction. Primary care physicians often serve as the first point of contact within the healthcare system and play a crucial role in coordinating and managing patients’ overall care [13]. The quality of this relationship can significantly impact patients’ overall healthcare experiences. Effective communication, empathy, and trust between patients and their physicians are essential components of a positive patient–physician relationship [14]. Patients who feel heard, understood, and respected by their physicians are more likely to be satisfied with their care and to follow medical advice.

Numerous studies have highlighted the importance of patient satisfaction as a determinant of healthcare utilization, adherence to treatment, and overall health outcomes. Satisfaction levels are influenced by various factors, including the physician’s communication skills, empathy, and competence, as well as the time spent with patients [15,16,17]. Additionally, the organizational aspects of healthcare services, such as accessibility, waiting times, and administrative support, also contribute significantly to patient satisfaction [18].

Understanding these dynamics is crucial for healthcare providers and policymakers to ensure that patient-centered care remains at the forefront of medical practice.

Educational institutions benefit both students and patients by providing training opportunities for students and by addressing patients’ dental care needs. It is crucial to assess patient satisfaction with the dental services offered in order to meet patient expectations, enhance patient cooperation, and maintain the dental institution’s performance [19]. It is also important to enable students to fulfill their clinical requirements promptly, as contented patients are more likely to comply and attend their visits. While dental clinics and hospitals prioritize patient satisfaction, educational settings prioritize student learning, which may occasionally lead to impaired patient satisfaction [20].

This study fills a need in the literature by providing an in-depth evaluation of dental treatment satisfaction among patients. Dental professionals and healthcare authorities can enhance the quality of care and patients’ experiences by recognizing and addressing the complex factors that contribute to satisfaction.

In order to fill these gaps, this study uses a mixed-methods approach to investigate in more depth the factors that contribute to dental patients’ satisfaction with their treatment. A more sophisticated understanding that is specific to different patient demographics and situations is needed since the weights assigned by patients to different satisfaction factors vary.

The purpose of this research is to add to the continuing efforts to improve dental patients’ experiences by providing a more complete picture of patient satisfaction through the use of quantitative and qualitative surveys with the help of a PSQ.

## 2. Materials and Methods

✓ Research design

This study aims to thoroughly investigate patient satisfaction with dental services. Due to the lack of dependable data on patient satisfaction in the multifaceted dental care system in Romania, our study focused on assessing patient satisfaction in a university dental clinic in Iasi and the factors that impact it. The research seeks to measure a wide range of factors affecting patient satisfaction by quantitative methods. The quantitative aspect includes a structured survey to measure the significance of different satisfaction factors in order to obtain a more profound understanding of patient experiences and perceptions.

✓ Survey instrument

The data were collected through a Patient Satisfaction Questionnaire consisting of 46 items (PSQ-46), which uses a 5-point Likert response scale ranging from strongly agree to totally disagree. The questions are divided into 6 dimensions that measure patients’ satisfaction toward physicians (20 items), access (8 items), nurses (4 items), appointments (4 items), and facilities (4 items), and a separate subscale of 6 items to measure overall satisfaction with the service provided by the practice. The PSQ was translated from English into Romanian following existing guidelines to maintain equivalence [21].

✓ Participants

The study sample consisted of patients who had received dental services within the last 10 months. Participants were recruited from the medical center of Gr.T.Popa Dental University, Iasi, Romania, and included patients from urban and suburban areas to ensure diversity in demographics and healthcare experiences. The inclusion criteria included adults aged 18 and above who had visited the dental clinic at least once in the past year. A total of 306 survey respondents were selected to represent various age groups, genders, and socioeconomic statuses. Their selection took into consideration the following inclusion and exclusion criteria:

Inclusion criteria

− Patients who had completed their dental treatment and who willingly consented to participate in the study.
− Patients aged eighteen years or older.

Exclusion criteria

− Patients unwilling to participate in the study and who were unable to provide informed consent.

✓ Data collection

A self-administered questionnaire was developed based on a review of the literature, which was then translated into the Romanian language and validated. The questionnaire included items on factors contributing to satisfaction and dissatisfaction like the frequency of dental visits, the quality of dental services, the role of communication with dental staff, information on waiting rooms, etc. [22]

The study received approval from the Institutional Review Board of UMF Gr.T.Popa, Iasi (No. 318/30.05.2023). Participants were informed about the study’s purpose, their rights, and confidentiality measures prior to data collection. Informed consent was obtained from all participants, and personal identifiers were removed from the data to ensure anonymity.

✓ Statistical analysis

Descriptive statistics were used to summarize the demographic information and responses to survey items. Inferential statistics, including regression analyses, were employed to identify significant predictors of patient satisfaction. 

Following data entry into an Excel spreadsheet, the statistical software for the social sciences, version 29, (Inc. Chicago, IL, USA) was used for processing.

Percentages, means, and standard deviations were calculated for the qualitative and quantitative data. Chi-square (X2) tests were performed to statistically analyze the qualitative data. A *p*-value of 0.05 was considered a significant difference.

## 3. Results

The study group consisted of 306 patients, who were predominantly women (58.5%), from urban areas (63.1%), and had a high school or university education (89.9%). Half of the patients were over 50 years old (50.3%), and a third (34.6%) were aged between 30 and 50 years. Additionally, half of the patients were employed (51.3%), and among the others, the majority were retirees (35.9%). Almost half of the patients had made between 5 and 10 visits to the dentist (44.8%), a third had made between 1 and 5 visits (30.4%), and a quarter of the patients (24.8%) had made more than 10 visits to the dentist (Table 1).

**Figure 1:**
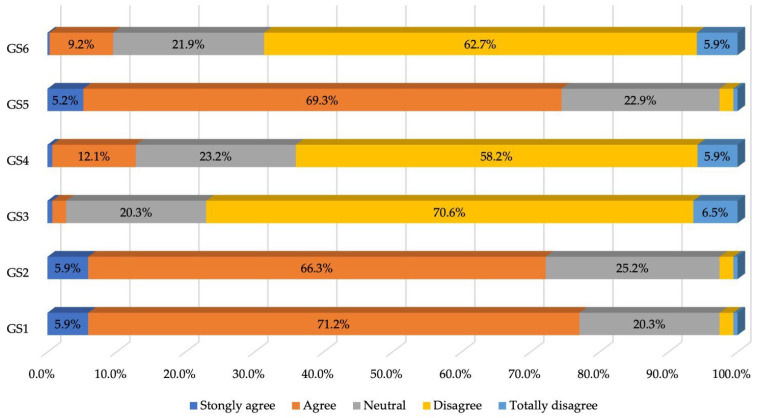
Percentage of responses to the first section of the questionnaire.

Questions with negative connotations were unfavorably received by patients, who expressed their partial or total disagreement with them; thus, it emerged that the vast majority of patients were satisfied with their doctor (77.1%). It can therefore be stated with certainty that the vast majority of patients declared a high overall satisfaction score, as shown in Table 2 and Figure 1.

This overall satisfaction score is reflected in the specific opinions of patients toward doctors, which was investigated in the second section of the questionnaire.

In full correlation with these results, it is noteworthy that negative or critical statements about doctors were rejected by patients, who expressed their agreement with these statements only at very low percentages. 

**Figure 2:**
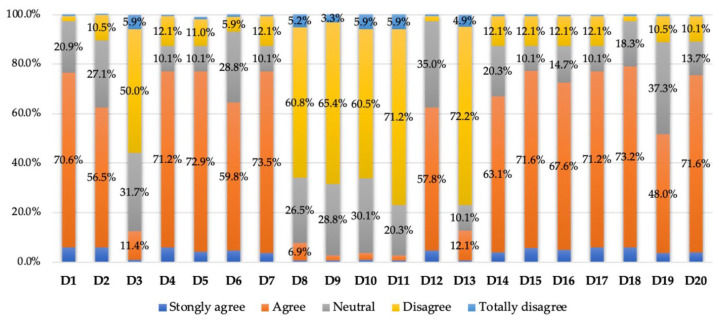
Percentage of responses to the second section of the questionnaire.

Therefore, patients believed that the doctor was indifferent to them only in isolated cases, and only a small percentage of patients (between 10 and 15%) were willing to claim that the doctor withheld some information from them or was not sufficiently empathetic toward their pathology and problems; however, it is clear that the vast majority of patients had favorable opinions about their communication with the doctor (see Table 3 and Figure 2).

**Figure 3:**
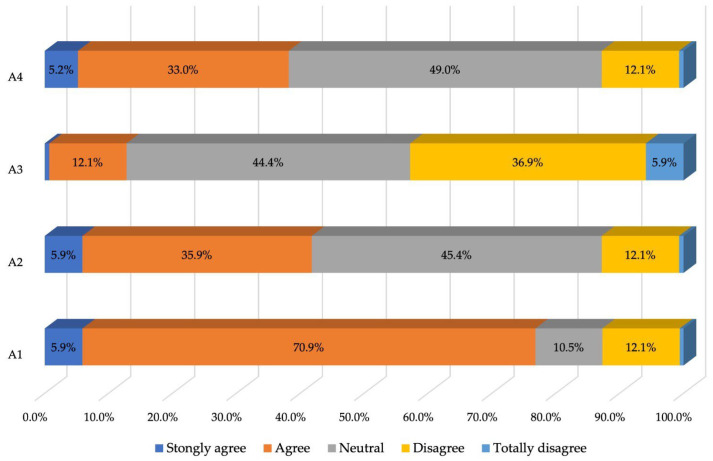
Percentage of responses to the third section of the questionnaire.

In general, patients considered it simple to schedule an appointment at the clinic when they wished, although only 40% of them maintained this opinion when it came to more specific situations, believing that they could easily schedule an appointment when they had a specific need or when they wanted to meet with a certain doctor (see Table 4 and Figure 3).

Patients’ evaluation of the accessibility in the clinic were slightly more reserved; thus, the predominance of patient agreement was only manifested in relation to items 6 and 7.

**Figure 4:**
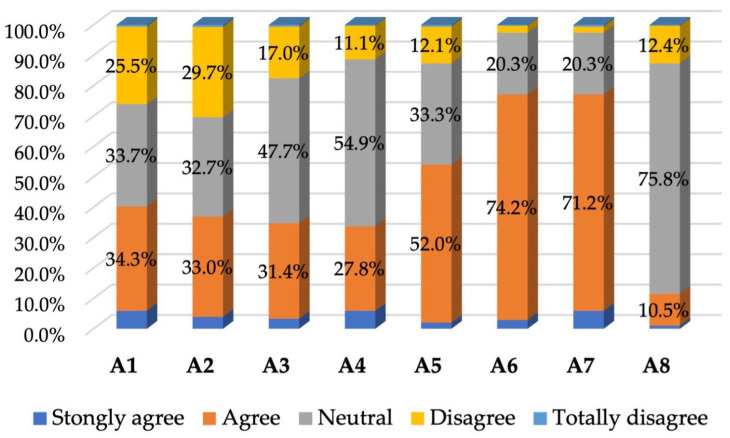
Percentage of responses to the fourth section of the questionnaire.

On the other hand, about half of the patients most likely faced the need to resolve emergencies and had a favorable interaction with the clinic in such situations, and about a third of the patients needed to discuss issues privately with the doctor and had a positive interaction, while another third probably did not face such situations, giving neutral responses to the items that targeted them; there was also a smaller percentage of patients who probably faced such situations but did not obtain the interaction they wished for from the doctor (see Table 5 and Figure 4).

Patients’ opinions toward nurses in dental offices were also generally favorable.

**Figure 5:**
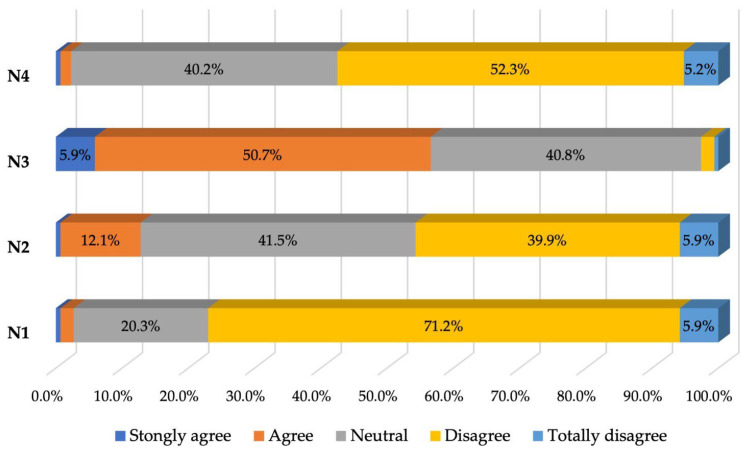
Percentage of responses to the fifth section of the questionnaire.

Very small percentages of patients had negative opinions about nurses: only 2.7% thought that they did not explain things carefully, only 2.3% felt that they made them feel like they were wasting their time, and 12.8% (a somewhat higher percentage) believed that the nurse did not always listen attentively when they talked about their problems (see Table 6 and Figure 5).

**Figure 6:**
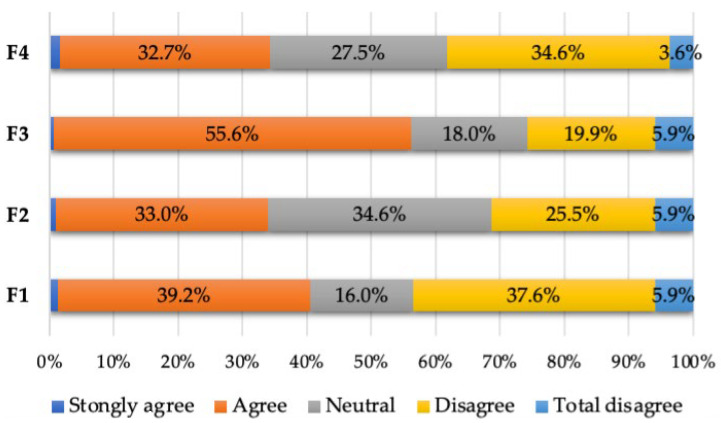
Percentage of responses to the sixth section of the questionnaire.

The final questions of the survey addressed the facilities offered by the dental office. The main issue reported by patients was the lack of seating in the waiting room—56.3% of them pointed out this aspect. It thus clearly emerges that patients’ unfavorable opinions primarily target the reduced capacity of the waiting room and, subsequently, the seats, which were perceived as being uncomfortable (see Table 7 and Figure 6).

Based on the responses recorded in the survey, we calculated, using arithmetic means, quantitative scores at the level of each patient, reflecting their general opinion toward the six dimensions evaluated by the survey, namely general satisfaction and opinions about doctors, the appointment system, the level of accessibility in the office, the behavior of the nurses, and the facilities offered.

These general scores have a variation range between 1 and 5, with a value of 1 meaning a completely favorable opinion and a value of 5 meaning a completely unfavorable opinion toward each of the investigated dimensions (to obtain such a result, the questionnaire items that targeted negative aspects were recoded, so that patient responses had uniform meanings across the entire survey). For the interpretation of the calculated quantitative scores, the corresponding interquartile ranges were used, as shown in Table 8. The overall satisfaction score of patients, total and comparative, by demographic characteristics is shown in Table 9.

## 4. Discussion

Donabedian outlines four distinct rationales for examining patient satisfaction. Satisfaction is a goal of care, a result of care, an outcome, and can enhance the impact of care by increasing patient compliance, and it is the patient’s evaluation of the care received.

Studies on patients’ satisfaction with their dental care have been around since at least the 1980s, but up until recently, researchers have mostly concentrated on how socio-demographic factors affect patients’ opinions of their dentists.

Researchers at multiple institutes have studied patient satisfaction with dental care. Patient satisfaction is influenced by various aspects beyond treatment quality, including facilities, personnel demeanor, and fundamental environmental requirements [23]. Most patients seeking treatment at dental training schools were between the ages of 30 and 40 in the majority of the studies [24]. More than half of the participants in this study were aged between 50–70 years (50.3%). This area requires development and should be addressed by the public health department and college management. Health camps should be organized to raise awareness of the services available at our institutions among the younger population in the region.

The notion of consumerism, which involves incorporating the patient’s perspective in the evaluation of services, has become more prominent in the last years. Patients can contribute to assessing the quality of oral health care by establishing standards of care, offering information for evaluation, and expressing satisfaction or dissatisfaction with the care received [25,26,27]. Most replies came from female patients (68%) because there was a higher volume of patients in the female division. This aligns with the findings of Naguib et al.’s study [28] but is in contrast to Habib et al.’s study, where the female response rate was 55.7% [29].

Patients’ happiness has been studied at several dental schools throughout different countries. The investigations revealed that the primary reason for seeking care in these clinics is due to the perceived high quality of service and the patients’ health concerns [30,31,32,33]. Patients who struggled to schedule appointments readily expressed a low level of satisfaction. The patients in the survey expressed a high level of satisfaction with their appointments (*p* = 0.034). They were also happy with the appointment selections that worked well with their schedule (35.9%). 

The reception desk and team typically handle appointments and are the initial point of contact for patients at the clinics. They play a crucial role in the team, and the high satisfaction levels noted in our study are promising. It is important to relay positive feedback to the reception team.

When it comes to the facilities, it has been discovered that patients are more satisfied when the facilities are nice, modern, and have comfortable waiting areas. Compared with studies conducted by Al-Refeidi et al. [34] and Mahrous et al. [35], this finding is significantly lower, but it is similar to a study conducted by Naguib et al. [28].

Several factors that affect dental patients’ happiness have been studied. One of the most important factors is the dentist’s communication abilities, which should include thorough explanations of procedures and treatments [9]. Bradshaw et al. found that patients are more satisfied when treatments are provided quickly and there is less waiting time [36]. Not only that, but the dental clinic’s physical setting, such as the comfort of the waiting rooms and the level of cleanliness, is also crucial [35]. 

One of the most important things a healthcare provider should have is good communication skills so that they can ensure that their patients are satisfied with the treatment they receive. A high degree of satisfaction has been linked to the dentist’s attitude and care for the patient’s demands, according to previous research [37,38]. Consumers’ willingness to use dentistry clinics is an area where little empirical data is available, according to Pinkerton et al. [39]. Despite widespread agreement that surveys of patients’ opinions are useful for gauging the quality of healthcare providers’ and facilities’ offerings, Holden et al. [40] found that researchers have paid surprisingly little attention to how satisfied dental patients are with their treatment. Othman and Abdel Razak [41] discovered that 45.6% of patients were satisfied with their dentists’ ability to explain treatment plans to them before they began. In the same manner, 70.6% of patients in our study reported being satisfied with how the doctor clearly explained everything before any treatment.

This might be because the study is taking place in a classroom setting, where teaching students how to properly communicate and engage with patients is a major emphasis. Patients dislike dentists who start treatments without explaining them, as pointed out by Hellyer [42].

A prior study stated that unhappiness with the way patients were treated by their dentists was frequently cited as the reason for switching dentists by 46% of the dentists polled. Patients reported being “unhappy with dentist” as the primary motivation for seeking out a new dentist in more current research [43].

The care our patients receive is of the utmost importance to us. This has led to very positive feedback from our patients about the treatment they received. Thus, 69.3% of our patients felt perfectly satisfied with how they were treated.

The Patient Satisfaction Questionnaire, consisting of 46 items (PSQ-46), is a widely used tool for measuring patient satisfaction with healthcare services [44]. It was developed by Ware and colleagues in the 1970s and 1980s as part of the Medical Outcomes Study and it was proved to be a robust tool for measuring patient satisfaction [45]. Its development was driven by the need for a reliable and valid instrument that could capture the multifaceted nature of patient experiences with healthcare services [46].

The quality of medical services can be assessed by considering the amount of patient satisfaction and the success rate of treatments. Attaining satisfactory patient outcomes and averting disease effects hinge on the crucial factor of satisfaction [47]. Furthermore, it serves as a primary objective of therapeutic activities and is a noteworthy measure of the standard of care.

The scale’s multidimensional approach, patient-centered focus, and established reliability make it a valuable tool for researchers and healthcare providers aiming to improve the quality of care and patient satisfaction [47].

Compared with other scales, PSQ-46 provides a more detailed analysis of patient satisfaction. For instance, SERVQUAL measures service quality across tangibles, reliability, responsiveness, assurance, and empathy, but it is more general and not healthcare-specific [48].

HCAHPS includes 29 items focusing on communication, responsiveness, environment, pain management, medication communication, discharge information, overall hospital rating, and willingness to recommend, primarily for public reporting and hospital comparisons in the U.S. [49].

PSSUQ focuses on system usability, with 16 items covering system usefulness, information quality, and interface quality, mainly for technology and electronic health records [50].

While SERVQUAL and HCAHPS are useful for broader service quality and standardized hospital comparisons, and PSSUQ is specific to technology usability, PSQ-46 stands out due to its detailed, patient-centered approach that is specifically designed for healthcare, making it particularly valuable for in-depth quality assessments.

One limitation of this study is that the five-point Likert scale can provide a wide range of responses. Also, the study relies on self-reported data from patients, which can be subject to response bias, including social desirability bias, where respondents may give answers that they believe are more socially acceptable.

On the other hand, there can also be recall bias, as patients’ satisfaction levels could be influenced by their memory of past experiences. More recent visits may be overemphasized compared with older ones.

With regard to geographic and demographic limitations, we can consider the fact that the study is confined to the northeast region of Romania, and the results may not be applicable to other regions with different healthcare systems, cultural contexts, or demographic profiles.

Addressing these limitations in future research could help improve the robustness and applicability of these findings in assessing patient satisfaction with oral healthcare services.

## 5. Conclusions

According to the study’s findings, participants were satisfied with the services, staff, treatment, and patient–dentist interaction in dental clinics run by the College of Dentistry of Iasi University. 

To maintain a high level of satisfaction and to make further improvements, patient satisfaction should be evaluated on a regular basis. Additionally, more qualitative research is needed to identify the psychological, behavioral, and social aspects that contribute to dental patients’ satisfaction with their treatment. 

Assessing patient satisfaction in the realm of dental service quality is crucial for enhancing service quality and accuracy, which would benefit both patients and dentists and, ultimately, improve public health.

## Figures and Tables

**Table 1:** Demographic characteristics of the study sample.

Study Group	Variables	*n*	%
gender	male	127	41.5
	female	179	58.5
age	18–30	46	15
	30–50	106	34.6
	50–70	154	50.3
occupation	employee	157	51.3
	no job	18	5.9
	retired	110	35.9
	student	21	6.9
domicile	urban	113	63.1
	rural	193	36.9
education	elementary school	31	10.1
	high school	153	50.0
	university	122	39.9

**Table 2:** Patient responses to the first section of the questionnaire—General satisfaction level (frequency distributions).

General Satisfaction:	Strongly Agree		Agree		Neutral		Disagree		Totally Disagree	
Answers	*n*	%	*n*	%	*n*	%	*n*	%	*n*	%
GS1: Patients receive the best care from the staff working in this office	18	5.9	218	71.2	62	20.3	6	2.0	2	0.6
GS2: I have absolute trust in doctors	18	5.9	203	66.3	77	25.2	6	2.0	2	0.6
GS3: I am not satisfied with my doctor	2	0.6	6	2.0	62	20.3	216	70.6	20	6.5
GS4: There are one or two things about this procedure that I am not satisfied with	2	0.6	37	12.1	71	23.2	178	58.2	18	5.9
GS5: I feel perfectly satisfied with how I am treated	16	5.2	212	69.3	70	22.9	6	2.0	2	0.6
GS6: I’ve been thinking about changing the office	1	0.2	28	9.2	67	21.9	192	62.7	18	5.9

**Table 3:** Patient responses to the second section of the questionnaire—Opinions about doctors (frequency distributions).

Doctors:	Strongly Agree		Agree		Neutral		Disagree		Totally Disagree	
Answers	*n*	%	*n*	%	*n*	%	*n*	%	*n*	%
D1: The doctor clearly explains everything before any treatment	18	5.9	216	70.6	64	20.9	6	2.0	2	0.7
D2: The doctor performs enough tests	17	5.6	173	56.5	83	27.1	32	10.5	1	0.3
D3: The doctor does not tell me enough about the treatment	3	1.0	35	11.4	97	31.7	153	50.0	18	5.9
D4: The doctor fully explains how the disease will affect my health	18	5.9	218	71.2	31	10.1	37	12.1	2	0.6
D5: The doctor carefully examines me	13	4.2	223	72.9	31	10.1	36	11.8	3	1.0
D6: The doctor is always interested	14	4.6	183	59.8	88	28.8	18	5.9	3	1.0
D7: The doctor always asks about how my disease affects my daily life	11	3.6	225	73.5	31	10.1	37	12.1	2	0.6
D8: Sometimes I feel like I didn’t get enough information from the doctors	2	0.6	21	6.9	81	26.5	186	60.8	16	5.2
D9: Sometimes the doctor makes me feel like I’m wasting his time	2	0.6	6	2.0	88	28.8	200	65.4	10	3.3
D10:I don’t feel confident discussing my problems with the doctor	3	1.0	8	2.6	92	30.1	185	60.5	18	5.8
D11: The doctor seems to want to get rid of me as soon as possible	2	0.6	6	2.0	62	20.3	218	71.2	18	5.9
D12: The doctor gives me every chance to talk about all my problems	14	4.6	177	57.8	107	35.0	6	2.0	2	0.6
D13: The doctor sometimes fails to appreciate how sick I am	2	0.6	37	12.1	31	10.1	221	72.2	15	4.9
D14: The doctor shows a real interest in my problems	12	3.9	193	63.1	62	20.3	37	12.1	2	0.6
D15: The doctor does everything necessary to arrive at a diagnosis	17	5.6	219	71.6	31	10.1	37	12.1	2	0.6
D16: The doctor always makes me feel at ease	15	4.9	207	67.6	45	14.7	37	12.1	2	0.6
D17: The doctor is very understanding	18	5.9	218	71.2	31	10.1	37	12.1	2	0.6
D18: The doctor knows when additional examinations are necessary	18	5.9	224	73.2	56	18.3	6	2.0	2	0.6
D19: Even when the doctor is busy, I am examined	11	3.6	147	48.0	114	37.3	32	10.5	2	0.6
D20: I don’t feel rushed when I’m with the doctor	12	3.9	219	71.6	42	13.7	31	10.1	2	0.6

**Table 4:** Patient responses to the third section of the questionnaire—Opinions about appointments (frequency distributions).

Appointments:	Strongly Agree		Agree		Neutral		Disagree		Totally Disagree	
	*n*	%	*n*	%	*n*	%	*n*	%	*n*	%
A1: It’s easy to get an appointment at a convenient time	18	5.9	217	70.9	32	10.5	37	12.1	2	0.6
A2: Appointments are easy to make whenever I need them	18	5.9	110	35.9	139	45.4	37	12.1	2	0.6
A3: It is often difficult to get a doctor’s appointment	2	0.6	37	12.1	136	44.4	113	36.9	18	5.9
A4: It’s easy to see the doctor of my choice	16	5.2	101	33.0	150	49.0	37	12.1	2	0.6

**Table 5:** Patient responses to the fourth section of the questionnaire—Opinions about accessibility (frequency distributions).

Accessibility:	Strongly Agree		Agree		Neutral		Disagree		Totally Disagree	
	*n*	%	*n*	%	*n*	%	*n*	%	*n*	%
A1: I feel it is easy to talk to my doctor over the phone	18	5.9	105	34.3	103	33.7	78	25.5	2	0.6
A2: The doctor is always available to give advice over the phone	12	3.9	101	33.0	100	32.7	91	29.7	2	0.6
A3: It is easy to get advice over the phone	10	3.3	96	31.4	146	47.7	52	17.0	2	0.6
A4: I am satisfied with the after-hours service	18	5.9	85	27.8	168	54.9	34	11.1	1	0.2
A5: The office has facilities to deal with emergencies that occur outside of opening hours	6	2.0	159	52.0	102	33.3	37	12.1	2	0.6
A6: Receptionists explain things to me clearly	9	2.9	227	74.2	62	20.3	7	2.3	1	0.2
A7: Receptionists ask patients the right questions	18	5.9	218	71.2	62	20.3	6	2.0	2	0.6
A8: I can speak to a receptionist privately if I wish	3	1.0	32	10.5	232	75.8	38	12.4	1	0.2

**Table 6:** Patient responses to the fifth section of the questionnaire—Opinions about nurses (frequency distributions).

Nurses:	Strongly Agree		Agree		Neutral		Disagree		Totally Disagree	
	*n*	%	*n*	%	*n*	%	*n*	%	*n*	%
N1: Nurses do not take care to explain things carefully	2	0.6	6	2.0	62	20.3	218	71.2	18	5.9
N2: The nurse does not always listen carefully when I talk about my problems	2	0.6	37	12.1	127	41.5	122	39.9	18	5.9
N3: Nursing is always very reassuring	18	5.9	155	50.7	125	40.8	6	2.0	2	0.6
N4: The nurse makes me feel like I’m wasting her time	2	0.6	5	1.6	123	40.2	160	52.3	16	5.2

**Table 7:** Patient responses to the sixth section of the questionnaire—Opinions about facilities (frequency distributions).

Facilities:	Strongly Agree		Agree		Neutral		Disagree		Totally Disagree	
	*n*	%	*n*	%	*n*	%	*n*	%	*n*	%
F1: The office could use some improvements	4	1.2	120	39.2	49	16.0	115	37.6	18	5.9
F2: The waiting room is uncomfortable	3	0.9	101	33.0	106	34.6	78	25.5	18	5.9
F3: There are not enough seats in the waiting room	2	0.6	170	55.6	55	18.0	61	19.9	18	5.9
F4: The seats in the waiting room are uncomfortable	5	1.5	100	32.7	84	27.5	106	34.6	11	3.6

**Table 8:** Interquartile ranges used.

		Range
1	Strongly agree	
2	Agree	1.81–2.60
3	Neutral	2.61–3.40
4	Disagree	3.41–4.20
5	Totally disagree	4.21–5.00

**Table 9:** The overall satisfaction score of patients—total and comparative—by demographic characteristics.

General Satisfaction									
		N	Media	Standard Error of the Mean	Std.dev	Min	Max	Mean	*p*-Value
**Total**		306	2.27	0.03	0.60	1.00	5.00	2.00	
**Gender**	female	179	2.25	0.05	0.67	1.00	5.00	2.00	
	male	127	2.33	0.04	0.47	1.83	4.00	2.16	*p* < 0.001
**Age**	18–30	46	2.13	0.13	0.88	1.00	5.00	2.00	
	30–50	106	2.39	0.05	0.59	1.00	4.00	2.00	*p* = 0.067
	50–70	154	2.21	0.04	0.50	1.00	4.83	2.16	
**Domicile**	rural	113	2.13	0.04	0.49	1.00	4.00	2.00	
	urban	193	2.30	0.04	0.64	1.00	5.00	2.00	*p* = 0.037
**Education**	elementary school	31	2.36	0.10	0.56	1.00	3.33	2.00	
	high school	153	2.14	0.04	0.55	1.00	4.00	2.00	*p* = 0.081
	university	122	2.42	0.05	0.63	1.83	5.00	2.08	
**Occupation**	employee	157	2.37	0.05	0.63	1.00	5.00	2.00	
	no job	18	2.31	0.12	0.53	2.00	3.33	2.00	*p* < 0.001
	retired	110	2.25	0.04	0.47	1.00	4.00	2.16	
	student	21	1.68	0.15	0.71	1.00	4.00	2.00	
**Appointments**	1–5	93	2.50	0.08	0.79	1.00	5.00	2.00	
	5–10	137	2.24	0.04	0.52	1.00	3.33	2.16	*p* = 0.034
	>10	76	2.05	0.03	0.32	1.00	2.67	2.00	

Mann–Whitney/Kruskal–Wallis tests were used. The significance level was set at *p* = 0.05.

## Data Availability

All data are available from the corresponding authors upon reasonable request.
